# A New Rat Model of Chronic Cerebral Hypoperfusion Resulting in Early-Stage Vascular Cognitive Impairment

**DOI:** 10.3389/fnagi.2020.00086

**Published:** 2020-04-15

**Authors:** Jinxin Wang, Chenyi Yang, Haiyun Wang, Dongxue Li, Tang Li, Yi Sun, Mingshu Zhao, Ji Ma, Wei Hua, Zhuo Yang

**Affiliations:** ^1^Department of Anesthesiology, The Third Central Clinical College of Tianjin Medical University, Tianjin Third Central Hospital, Nankai University Affinity the Third Central Hospital, Tianjin Institute of Hepatobiliary Disease, Tianjin Key Laboratory of Extracorporeal Life Support for Critical Diseases, Tianjin, China; ^2^Medical College of Nankai University, Nankai University, Tianjin, China; ^3^Tianjin Research Institute of Anesthesiology, Tianjin, China

**Keywords:** vascular cognitive impairment, chronic cerebral hypoxia, cognitive function, bilateral carotid artery stenosis, anisotropy

## Abstract

**Objective:**

Currently, most models of vascular cognitive impairment are established by occluding the carotid arteries uni- or bilaterally to reduce the cerebral blood flow mimicking chronic cerebral hypoxia. Due to the sudden blood flow interruption, a gradual narrowing of the carotid artery cannot be completely imitated. This paper aims to establish a bilateral carotid stenosis model with mild cognitive dysfunction and mild white matter changes to simulate patients with vascular predementia.

**Methods:**

Aged Wistar rats (18 months old) underwent either bilateral common carotid artery stenosis (BCAS) or occlusion (BCAO) surgery or a sham operation (control group). The cerebral blood flow in the frontal cortex was measured using Doppler flowmetry. Thirty days after surgery, cognitive function impairments were determined with the Morris water maze; cerebral magnetic resonance imaging was performed to detect changes in fractional anisotropy to assess white matter injuries, and histological studies were performed.

**Results:**

The aged rats in the BCAS group showed a more gradual cerebral blood flow reduction and a lower mortality rate (11%) compared to rats in the BCAO group. The water maze test revealed a more marginal impairment affecting spatial learning and memory in rats with BCAS than in rats with BCAO. Diffusion tensor imaging detected white matter injuries in the hippocampus and cerebral cortex of BCAS rats. Particularly, a small portion of nerve fibers of the lateral somatosensory cortex was significantly different between rats of the BCAO and BCAS groups. In the BCAS group, the microscopic structure of the hippocampal CA1 region changed slightly after 30 days and sustained a slight mitochondrial crista crack. Fluorescence staining indicated that the number of GFAP-positive cells was increased in rat brains of the BCAS group, and this phenomenon was even more pronounced in the BCAO group. The hnRNPA2/B1 and GABA_A_R-α1 expression levels were significantly decreased in the hippocampus of rats with BCAS compared to those of controls.

**Conclusion:**

Severe bilateral carotid stenosis induced mild cognitive dysfunction and slight structural changes in the brains of aged rats. Thus, a chronic cerebral hypoperfusion model was successfully established.

## Introduction

A chronic decline in sustained cerebral blood flow is the main mechanism underlying the development of vascular cognitive impairment (VCI) (Kazumata et al., 2019; Kim et al., 2019; Mansour et al., 2019; McKetton et al., 2019). Thus, animal models of chronic cerebral hypoperfusion are most commonly used to study VCI (Duncombe et al., 2017; Mansour et al., 2018; Washida et al., 2019).Therefore, our future studies aim to combine age factors with chronic cerebral hypoperfusion allowing the exploration of changes in the white matter and cognitive functions in elderly rats under different degrees of hypoperfusion and to establish a new VCI model of elderly rats with bilateral common carotid artery (CCA) stenosis (BCAS).

There are two risks of VCI in our model, which is close to elderly patients with asymptomatic carotid stenosis in clinical practice. In recent, an increasing number of elderly patients are presenting with asymptomatic carotid stenosis accompanied by varying degrees of cognitive dysfunction (Elhfnawy et al., 2019; Gray et al., 2019; Nickel et al., 2019; Seo et al., 2019). Pharmacological treatments such as propofol and sevoflurane (Shmelev and Neimark, 2013), as well as non-pharmacological therapy options (Ogutu et al., 2014; Lal et al., 2017; Gray et al., 2019) such as surgical treatment, should be used with caution to reduce the incidence of concurrent cognitive impairment. Therefore, it is essential to establish novel VCI models characterized by simplicity, stability, and low mortality to enable drug tests that evaluate their potential efficacy before clinical trials.

According to established standards in the field (Venkat et al., 2015; Hainsworth et al., 2017), the main purpose of establishing an animal model of vascular cognitive impairment is to replicate the pathological process of human cognitive impairment. In the present study, the main goal was to replicate the pathological process of white matter damage; this process combines the decline in vascular circulation and the age-dependent narrowing of the carotid arteries. Therefore, in this study, the changes in cerebral blood flow were evaluated and diffusion tensor imaging (DTI) sequences of magnetic resonance imaging (MRI) data were used to detect changes in the white matter. Additionally, glial activation and hippocampal cell damage were measured, and cognitive function changes were evaluated using the Morris water maze. The study by Mizukami et al. revealed that heterogeneous nuclear ribonucleoprotein (hnRNP) A2/B1 is elevated in the CA2 region of patients with mild cognitive impairment (Mizukami et al., 2005). By contrast, its expression is decreased in dementia (Kolisnyk et al., 2016). Therefore, whether hnRNPA2/B1 is equally elevated in the animal model or not is the present criterium to screen models of chronic hypoxia. The current study refers to previously established models and standards and compares our model with carotid occlusion models of the same age to determine the differences and advantages of the newly established model.

## Materials and Methods

### Animals

A total of 106 aged male Wistar rats (18 months, 500 ± 50 g) were randomly divided into three groups (BCAS, n = 30/34 [number of surviving animals/total number]; BCAO, bilateral CCA occlusion, n = 30/40; and sham operation, n = 30/32). The rats surviving postoperatively were randomly assigned to transmission electron microscope (n = 6), immunofluorescence (n = 6), and western blotting (n = 6) experiments or examined using MRI (n = 6) and the Morris water maze test (n = 6).

All animals were purchased from the Laboratory Animal Centre of the Military Medical Science Academy of the Chinese People’s Liberation Army. All animals were housed in cages in a temperature- and humidity-controlled room on a 12-h light-dark cycle with standard food and water *ad libitum*. All surgical procedures were approved by the Institutional Animal Care and Use Committee of Tianjin Medical University. Rats were treated according to the guidelines of the National Institutes of Health Guidelines and the UK Animals (Scientific Procedures) Act (1986) for the Care and Use of Laboratory Animals.

### Surgical Procedures in the Bilateral Common Carotid Artery Stenosis and Occlusion Rat Models

**FIGURE 1:**
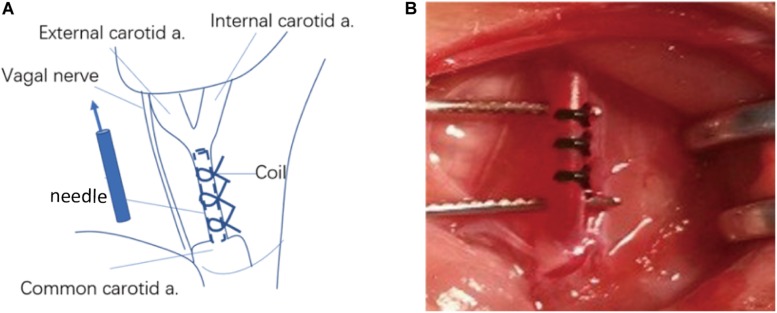
Schematic diagram of common carotid artery stenosis operation. **(A)** Operative procedure of carotid artery stenosis and anatomical structure around the common carotid artery. **(B)** Common carotid artery after constriction. a. : artery.

BCAS and bilateral CCA occlusion (BCAO) surgeries were performed to explore the differences between the two rat models of vascular hypoperfusion-induced dementia. After an intraperitoneal injection of 10% thiotetrabarbital (100 mg/kg), a midline neck incision was made to expose and dissociate the bilateral CCAs. For the 34 rats of the BCAS group, a syringe needle (diameter 0.45 mm) was tightly tied to the CCA bilaterally at 1.5 cm from the bifurcation of the internal and external carotid arteries. After ensuring that the slipknot was firmly fixed, the needle was carefully removed, and the wound was sutured. In the BCAO group, the CCAs of 40 rats were exposed, isolated, and clipped with metal aneurysm clips. The additional 30 rats in the control group were sham-operated; i.e., the bilateral CCAs were only exposed and dissociated (Figure 1).

No more than 1 ml of buprenorphine (0.3 mg/kg in saline, intraperitoneal) was administered for supplemental analgesia. During surgery, the body temperature of the animal was maintained at 37.0 ± 0.5°C using a heat lamp and heat blankets controlled by the feedback from a small rectal temperature probe (small animal constant temperature monitoring system, DL Naturegene). Penicillin (10^5^ UI, intraperitoneal injection) was administered intraperitoneally for 5 days after the operation to prevent infections. Non-invasive blood pressure monitors were used to measure the blood pressure via the tail arteries at 5 min before and after surgery. Baseline cerebral blood flow (CBF) recordings were obtained just before and at 2 h, 1 day, 3 days, 7 days, 14 days, and 30 days after surgery. The CBF values are expressed as percentages of the baseline value. On the 30^th^ day after surgery, the rats were used in histological, morphological, and MRI tests.

### Morris Water Maze Test

Rats (n = 6) performed the Morris water maze test 30 days after surgery to evaluate spatial learning and memory. The Morris water maze consisted of a large circular pool with a diameter of 180 cm and a height of 45 cm. It was filled with water (25 ± 1°C) to a depth of 30 cm, and a platform with a diameter of 12 cm was installed at a height of 25 cm. The pool was divided into four equal quadrants using two perpendicular threads. On the first day, the rats could swim freely for 2 min in the water pool. The spatial training test was performed on days 2–5 and repeated four times in four different quadrants at two fixed time points per day. The position of the platform remained the same throughout the training period. A computer was used to record the escape latency required for the rats to find and climb onto the transparent platform after being placed in the water. On the sixth day, the spatial search test was conducted where the time required to swim to the platform from the quadrant contralateral to the platform was recorded.

### Ultrasound Recordings

An echocardiograph (Vivid 7, GE Medical Systems, Horten, Norway) equipped with a 12-MHz linear transducer (12L) was used. Data were transferred online to an ultrasound image workstation for subsequent analysis (PC EchoPAC, GE Medical Systems). Ultrasound data comprised Doppler spectral recordings of the right and left CCAs in rats of the BCAS and BCAO groups. Laser Doppler flowmetry (PeriCam PSI, PERIMED AB) was used to detect the CBF. The region of interest in the frontal cortex was consistently set as a circle with 3 mm diameter at 2 mm posterior and 3 mm lateral to bregma. CBF was expressed as the percentage of the baseline level. Ultrasound recordings were repeated in each rat before and 2 h after surgery as well as 1, 3, 7, 14, and 30 days after surgery (n = 6).

### Magnetic Resonance Imaging Experiments

For MRI scans, the animals were fixed on an animal bed (Bruker, Ettlingen, Germany), the head was fixed using dental strips and dental pads, and the rats were anesthetized by continuous intranasal delivery of 2% isoflurane. The respiratory and heart rates as well as the body temperature of the animals were recorded to maintain a body temperature of 37.0 ± 0.5°C. Blood oxygenation was detected using a fiber-optic pulse oximetry sensor located on the foot of the animal. At the end of the MRI session, the rat was sacrificed under anesthesia by cervical dislocation.

### Image Acquisition

A Bruker Pharmascan 7 T imaging system (Bruker, Ettlingen, Germany) was used. In each animal, the following sequences were used: 1) two-dimensional Turbo Rapid Imaging with Refocused Echoes T2-weighted images (in three orthogonal directions) while positioning with the following scanning parameters: repetition time 5,000 ms; echo time 36 ms; 40 pixel 0.7 mm; field of view 30 × 30 mm^2^; pixel size 0.12 × 0.12 mm^2^ and 2) two-dimensional DTI scans using echo-planar imaging sequences with the scan parameters: repetition time 7,000 ms; echo time 22 ms; 20 coronal slices 0.8 mm; slice gap 0.1 mm; b-value 1,000 s/mm^2^; diffusion gradient pulse duration δ 4 ms; diffusion gradient interval Δ12 ms. Left and right frequency codes were used to extend the diffusion sensitization gradient in 45 optimal directions with a flip angle of 90°. Fifteen b0 images (b = 0 s/mm^2^ and 5 b0 images per 20 diffusion-weighted images) were acquired. The total acquisition time was approximately 20 min.

### Diffusion Tensor Imaging Data Analysis

The statistical parametric mapping-compatible DTI toolbox was used in the statistical process for data calibration and voxel-based analysis. The DTIStudio software^1^ was used to compensate for motion, and the diffusion-weighted image was linearly registered. DTIStudio calculated the fractional anisotropy (FA) values. All normalized FA images were cropped to 1.0 × 1.0 × 1.5 mm^3^ voxels (after zooming) for Gaussian smoothing (2.0 × 2.0 × 4.0 mm^3^ voxels [Gaussian kernel]), and a voxel-based analysis was performed. In the entire brain, 20 + voxel clusters with significant differences in FA between groups (P < 0.005) were marked. The statistical analysis was performed using the SPM8 and spmratIHEP toolboxes^2^. The data were spatially normalized to allow for voxel comparisons between animals. Data were compared between groups using an analysis of variance (ANOVA). The critical t-value was determined with 3.169 [t(10) = 3.169, P = 0.005].

### Transmission Electron Microscopy

To investigate the effects of chronic cerebral hypoxia induced by CCA stenosis or atresia on hippocampal cell nuclei, mitochondria, endoplasmic reticula, and cell membranes, transmission electron microscopy was performed on sections from the hippocampal CA1 area of rats 30 days after surgery. The rats were perfused with 2% paraformaldehyde and 2.5% glutaraldehyde. The hippocampus was then removed and cut into sections with a thickness of 1 × 1 × 5 mm^3^ for transmission electron microscopy. These sections were stained for 5 min in lead citrate, rinsed, post-stained for 30 min in uranyl acetate, rinsed again, and dried. Electron microscopy was performed at 60 kV using a Philips Morgagni transmission electron microscope equipped with a charged-couple device camera. Images were collected at 3,000–15,000 × magnification.

### Immunofluorescence Assay

Coronal hippocampal slices (10 μm thick) were paraffin-embedded and placed on slides. Primary antibodies included anti-hnRNPA2/B1 antibodies (1:200; Santa Cruz Biotechnology, catalog #sc-10035), anti-microtubule-associated protein 2 antibodies (1:200; PhosphoSolutions, catalog #583-FOX3), anti-gamma-aminobutyric acid receptor (GABA_A_) subunit α1 antibodies (1:500; Abcam, catalog #ab7260), and anti-glial fibrillary acidic protein (GFAP) antibodies (1:500; Abcam, catalog #ab4674). Sections were visualized using a Zeiss LSM 510 Meta confocal system equipped with 10 × and 40 × objectives, as well as a 488 nm Argon laser and a 594 nm helium-neon laser for the excitation of the fluorophores. Data were analyzed using Image-Pro Plus 6.0 software (Media Cybernetics, United States). The region of interest value was determined as the mean value in four random fields of the perinecrotic hippocampal CA1 region in four non-continuous sections. The imaris^3^ and ImageJ Fiji is just ImageJ^4^ was used to evaluate the morphology of astrocytes and Image rendering, which of processing method accord to the reference (Daniel-Christoph et al., 2013; Young and Morrison, 2018).

### Western Blotting Assay

Hippocampal tissue samples were obtained on post-surgical day 30. Membrane protein isolation was performed using the Mem-PER Plus^TM^ membrane protein extraction kit (Thermo Fisher Scientific, United States) according to the manufacturer’s instructions. The following primary antibodies were used: polyclonal rabbit anti-GABA_A_-α1 subunit (1:500; Abcam, catalog #ab7260), monoclonal mouse anti-hnRNPA2/B1 (1:200; Santa Cruz Biotechnology, catalog #sc-10035), monoclonal mouse anti-amyloid β (1:2,000, Sigma-Aldrich, United States), and monoclonal mouse anti-β-actin (1:10,000, Proteintech, United States). Image-Pro Plus was used for image acquisition and analysis.

### Statistical Analysis

All data are presented as the mean ± the standard deviation (SD). The performance in the Morris water maze test was analyzed using a two-way repeated measure ANOVA followed by the Bonferroni *post hoc* test. One-factorial ANOVA was used to compare physiological parameters. The body weight, mean arterial blood pressure, and CBF values were analyzed by repeated measure ANOVA followed by the Dunnett *post hoc* test. Mortality is presented in Kaplan-Meier graphs. All other data were analyzed using the one-way ANOVA followed by the Bonferroni *post hoc* test. P < 0.05 was considered statistically significant.

## Results

### Morris Water Maze Test

The results of the place navigation experiments showed that the time required to reach the platform (average escape latency) in the BCAS and BCAO groups was longer than in the control group (P < 0.05 *vs.* the control group) on the second to the fifth day of the experiment. Moreover, BCAO rats took longer to find the platform compared to BCAS rats (P < 0.05 *vs.* the BCAS group). Moreover, the frequency of original platform crossings within 60 s was reduced in both groups compared to the control group (P < 0.05). However, the frequency of crossings decreased when the rats were treated with BCAO compared with the BCAS group (P < 0.05 *vs.* the BCAS group), indicating poor memory. Hence, the memory impairment of rats in the BCAO group is more serious than that in the BCAS group (Tables 1, 2).

**TABLE 1:** Comparisons of the average escape latencies in the place navigation test.

Group	n	Day 2	Day 3	Day 4	Day 5
Control	6	32.77 ± 6.51	22.87 ± 4.51	20.42 ± 2.62	10.87 ± 1.63
BCAS	6	44.37 ± 6.61^∗^	33.86 ± 3.32^∗^	24.86 ± 3.42^∗^	15.86 ± 2.34^∗^
BCAO	6	47.87 ± 7.62^*#^	38.87 ± 5.63^*#^	34.32 ± 2.41^*#^	33.87 ± 2.24^*#^

*Data are expressed as the mean ± SD. ^∗^*P* < 0.05 compared with the control group; ^#^*P* < 0.05 compared with the BCAS group. BCAS, bilateral common carotid artery stenosis; BCAO, bilateral common carotid artery atresia; SD, standard deviation.*

**TABLE 2:** Comparisons of the times over the hidden platform and percentages of the swimming time to the platform in the spatial probe test.

Group	n	Percentage of the swimming time to the platform in the spatial probe test	Time over the hidden platform
Control	6	44.87 ± 4.36	3.80 ± 0.84
BCAS	6	34.34.87 ± 2.13^∗^	2.60 ± 0.54^∗^
BCAO	6	20.13 ± 5.23^*#^	1.60 ± 0.89^*#^

*Data are expressed as the mean ± SD. ^∗^*P* < 0.05 compared with the control group; ^#^*P* < 0.05 compared with the BCAS group. BCAS, bilateral common carotid artery stenosis; BCAO, bilateral common carotid artery atresia; SD, standard deviation.*

### Mortality Rates in the Bilateral Common Carotid Artery Stenosis and Occlusion Groups of Aged Rats

All surgical procedures in the BCAS and BCAO groups were accomplished within 15–20 min (except for an interval of 30 min). The preoperative body weights and diameters of the distal CCAs did not differ significantly among the experimental groups. The mean arterial blood pressure values of the surviving rats did not change significantly for any postoperative interval up to 30 days compared with the sham-operated controls.

**FIGURE 2:**
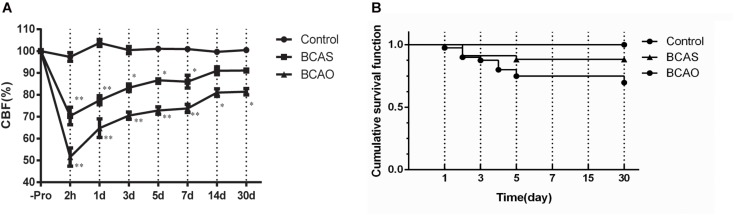
The survival rates (**A**) and CBF values (**B**) in each group after surgery. The data are presented as the mean ± SD and expressed as percentages of the preoperative value. *P < 0.05 and **P < 0.01 compared with the control group using Dunnett’s multiple comparison test. CBF, cerebral blood flow; BCAS, bilateral common carotid artery stenosis; BCAO, bilateral common carotid artery occlusion; SD, standard deviation.

In the BCAS group, the mortality rate was 11% (4/34). By contrast, 25% (10/40) died in the BCAO group within 14 postoperative days; most of which were found to have cerebral infarctions (Figure 2A). In the sham-operated controls, all rats survived until euthanized on day 30. In the BCAS group, all rats regained consciousness within a few hours but occasionally showed transient ptosis. However, no apparent motor weakness was observed. By contrast, in the BCAO group, 35% (13/40) of the rats did not regain consciousness within the first 3 h postoperatively, whereas 30% (12/40) exhibited rolling or circling movements lasting 2–6 h after awakening, and 20% (8/40) showed severe akinesia with a squatting posture.

### Cerebral Blood Flow Changes in the Bilateral Common Carotid Artery Stenosis and Occlusion Groups of Aged Rats

For the analysis of the CBF changes, only values of the surviving rats were included. In the control group, the mean CBF values (mean ± SD) after a sham operation varied from 97.31 ± 1.99% to 103.71 ± 1.55% without any significant changes between time intervals (one-factorial ANOVA, 0.067). By contrast, the CBF values decreased significantly from the preoperative baseline after the surgery in both the BCAS and BCAO groups. At 2 h, there was a significant reduction in the CBF values to 70.33 ± 4.00% in the BCAS group and to 51.51 ± 4.18% in the BCAO group. On day 1, the CBF values began to recover but remained significantly lower in both groups until day 14, compared with the control group. On day 28, the CBF values were still decreased in the BCAO group. Intergroup differences in CBF values were detected among the BCAS, BCAO, and control groups (Figure 2B).

### Progressive Microstructural Alterations in Aged Rats of the Bilateral Common Carotid Artery Stenosis and Occlusion Groups

**FIGURE 3:**
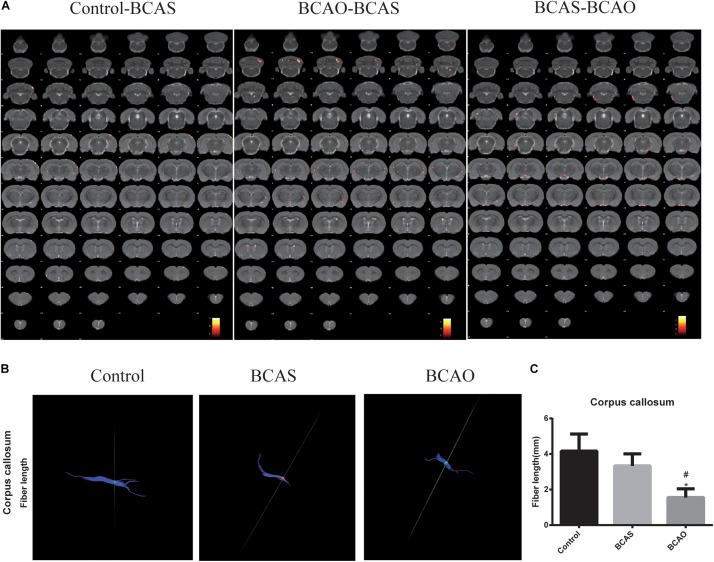
**(A)** Voxel-based statistical analysis of the diffusion tensor imaging parameter FA. **(B,C)** The change of white matter neural fiber **(B)** and fiber length in corpus callosum. The significant main effects of the phenotype are shown for the mean diffusivity. Results are displayed on an anatomical template with a visualization threshold of false discovery rate with *P* < 0.005 and a minimum cluster size of 20 voxels. The color scale indicates f-values, with yellow indicating a larger difference between the conditions investigated. Control-BCAS means that the FA value in BCAS rats is lower than in controls. BCAS-BCAO means that the FA value in BCAS rats is higher than in BCAO rats. The strongest effects according to the voxel-based analysis are displayed in the graphs. Data are expressed as the mean ± SD (*n* = 6/group). ^∗^*P* < 0.05 compared with the control group; ^#^*P* < 0.05 compared with the BCAS group. FA, fractional anisotropy; BCAS, bilateral common carotid artery stenosis; BCAO, bilateral common carotid artery occlusion; SD, standard deviation.

To assess whether microstructural changes can be observed after BCAS or BCAO surgery in aged rats, a voxel-based DTI parametric map analysis was performed. As shown in Figure 3, several clusters of FA values in BCAS animals were found to be significantly altered compared to controls. More specifically, reduced FA values are present in the ventricular region of BCAS animals, whereas the FA values are significantly elevated in their primary and somatosensory cortexes. BCAS animals were shown to have a strong FA value reduction in a large bilateral cluster consisting of the ventral portion of the external capsule extending into the entorhinal cortex. In the cortex, the FA values are lower in BCAS animals compared to control animals (Table 3). Increased FA values are present in a cluster containing the body and the back of the external capsule in BCAS animals. Additionally, a small portion of the lateral somatosensory cortex presented higher FA values in the BCAO group compared to the BCAS group.

**TABLE 3:** Regions with FA value decreases in the brains of BCAS rats compared with control.

Brain area	Left/Right	KE	Z-value	P-value	MNI coordinates (mm)		
					X	Y	Z
External capsule	right	68	3.8300	0.0000	7	3	1
M1	right	96	3.8100	0.0000	3	1	15
Cpu	left	215	3.6400	0.0000	4	1	−2
S1-FL	right	76	3.4300	0.0000	3	8	6
S1-Sh	right	52	3.4300	0.0000	4	0	8
Cpu	left	54	3.3200	0.0000	3	−1	0

*MNI, Montreal Institute of Neurology (X, Y, and Z coordinates locate anatomical structures). FA, fractional anisotropy; BCAS, bilateral common carotid artery stenosis; KE, number of continuum elements; Z-value, activation cluster peak value; M1, primary motor cortex; Cpu, Nucleus Caudate Putamen; S1FL, primary somatosensory cortex forelimb; S1SH, primary somatosensory cortex shoulder.*

### Ultrastructural Changes in the Bilateral Common Carotid Artery Stenosis and Occlusion Groups of Aged Rats

**FIGURE 4:**
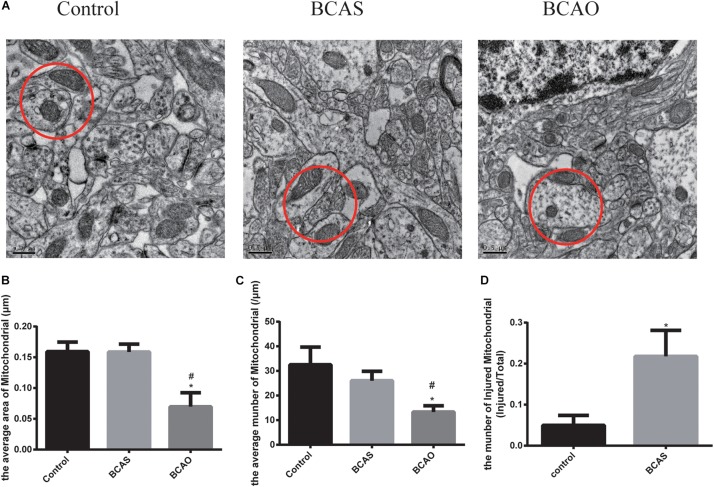
Electron microscopy data for the hippocampal CA1 area in the three experimental groups. **(A)** Red circles indicate mitochondria. The average area **(B)** and total number of mitochondria **(C)** in the hippocampal CA1 area 30 days after surgery are depicted. **(D)** The number of injured mitochondria (mitochondrion with crista broken) in the control and BCAS groups. Data are expressed as the mean ± SD (*n* = 6/group). *P < 0.05 compared with the control group, ^#^*P* < 0.05 compared with the BCAS group; BCAS, bilateral common carotid artery stenosis; BCAO, bilateral common carotid artery occlusion; SD, standard deviation.

Transmission electron microscopy revealed that the ultrastructure in animals of the experimental groups had changed. The neurons in the hippocampal CA1 area of the BCAS group were damaged, and the nucleolus became smaller; also, the rough endoplasmic reticulum, Golgi apparatus, and mitochondria were swollen, and apoptotic bodies were occasionally found. In the BCAO group, neuronal damage in the hippocampal CA1 area was aggravated, the endoplasmic reticulum and Golgi apparatus appeared even more swollen than in the BCAS group, and apoptotic bodies were more frequently observed. In the control group, neuronal nucleoli were normal, the endoplasmic reticulum and mitochondria did not appear swollen, and ribosomes were abundant (Figure 4).

### Hippocampal Expression of Heterogeneous Nuclear Ribonucleoprotein A2/B1 and Gamma-Aminobutyric Acid Receptor Subunit α1 Changes 30 days After Bilateral Common Carotid Artery Stenosis and Occlusion Surgery

**FIGURE 5:**
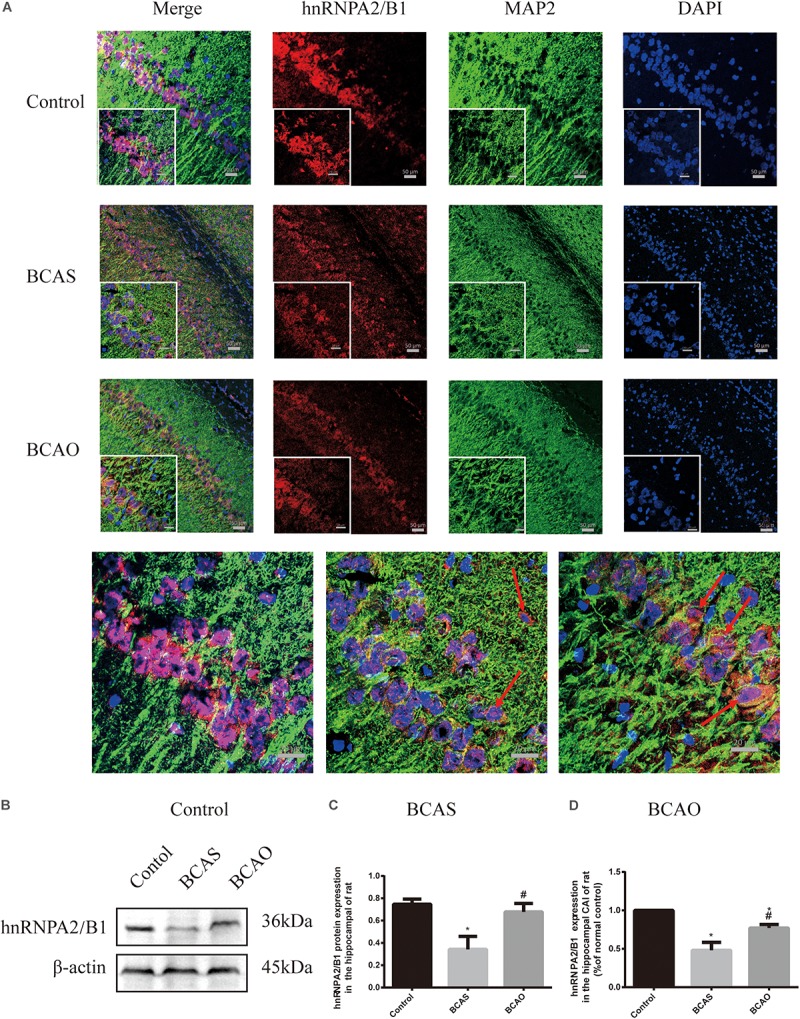
Expression of hnRNPA2/B1 at 30 days after surgery. Protein detected by immunofluorescence in the hippocampal CA1 region (**A and C**). Hippocampal hnRNPA2/B1 expression detected by western blotting (**B and D**). Data are expressed as the mean ± SD (*n* = 6/group). ^∗^*P* < 0.05 compared with the control group; ^#^*P* < 0.05 compared with the BCAS group; BCAS, bilateral common carotid artery stenosis; BCAO, bilateral common carotid artery occlusion; hnRNPA2/B1, heterogeneous nuclear ribonucleoprotein A2/B1; SD, standard deviation; MAP2, Microtubule associated protein 2; DAPI, 4’,6-diamidino-2-phenylindole.

**FIGURE 6:**
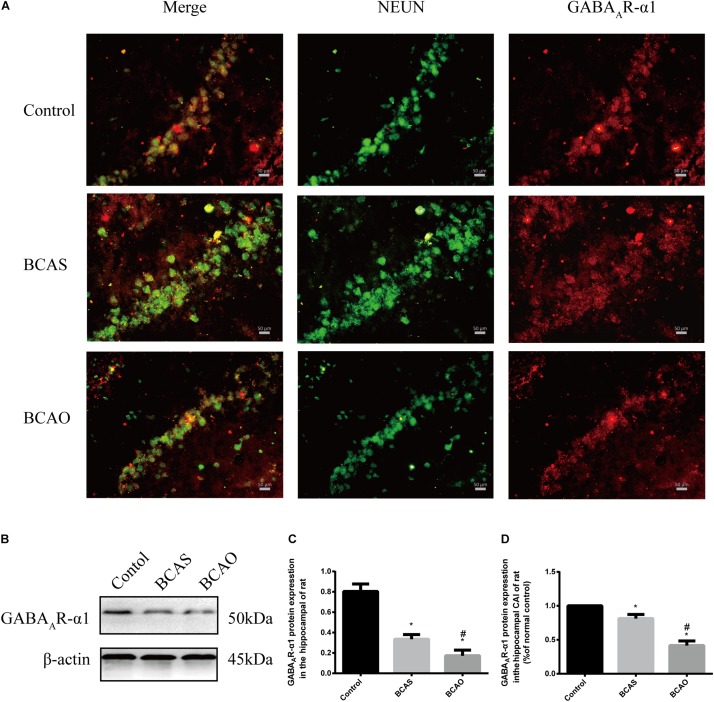
GABA_A_R-α1 expression in the hippocampal CA1 area at 30 days after surgery. Protein detected by immunofluorescence (**A and C**). GABA_A_R-α1 expression in the rat hippocampus detected by western blotting (**B and D**). Data are expressed as the mean ± SD (*n* = 6/group). ^∗^*P* < 0.05 compared with the control group; ^#^*P* < 0.05 compared with the BCAS group. BCAS, bilateral common carotid artery stenosis; BCAO, bilateral common carotid artery occlusion; GABA_A_R-α1, gamma-aminobutyric acid receptor subunit α1; SD, standard deviation.

The most interesting result was the decreased hnRNPA2/B1 expression in the BCAS group compared to the control group, whereas the hnRNPA2/B1 expression level in the BCAO group was elevated compared to the BCAS group. In the BCAS group, hnRNPA2 expression was elevated in the cytoplasm (Figure 5). Both the BCAS and BCAO groups presented robust decreases in GABA_A_R-α1 levels in the hippocampus; this change was more severe in the BCAO group (Figure 6).

### Astrocyte Proliferation and Hippocampal Neuronal Apoptosis in the Hippocampal CA1 Region of Rats Exposed to Bilateral Common Carotid Artery Stenosis or Occlusion 30 days After Surgery

**FIGURE 7:**
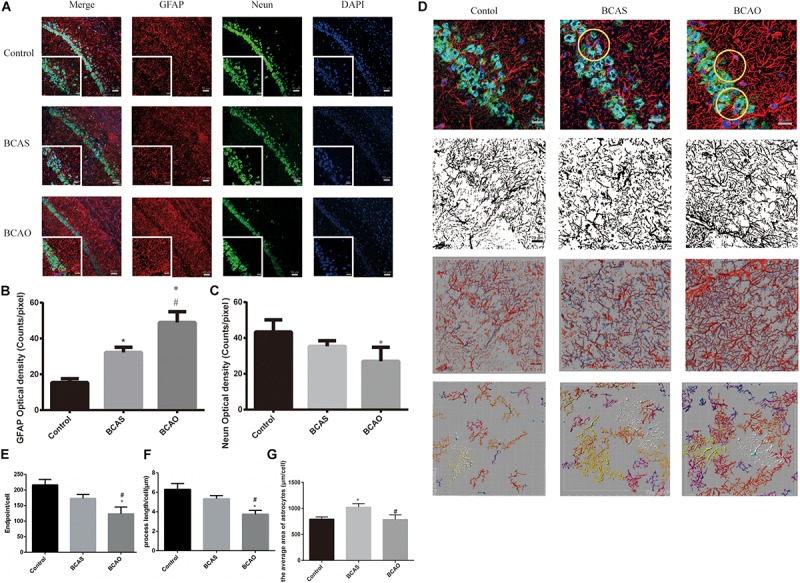
Astrocyte proliferation and neuronal apoptosis in the hippocampal CA1 region of BCAS and BCAO rats (**A**). GFAP and NeuN expression in the hippocampal CA1 area 30 days after surgery (**B and C**). The endpoint, the process length and average area of astrocytes. **(D–G)** Data are expressed as the mean ± SD (*n* = 6/group). ^∗^*P* < 0.05 compared with the control group; ^#^*P* < 0.05 compared with the BCAS group. BCAS, bilateral common carotid artery stenosis; BCAO, bilateral common carotid artery occlusion; SD, standard deviation; GFAP, glial fibrillary acidic protein; DAPI, 4’,6-diamidino-2-phenylindole.

The NeuN immunofluorescence images show that in the CA1 region of the rat hippocampus, the number of surviving neurons was decreased in the BCAS and BCAO groups compared with the control group (P < 0.05). The lesions in the CA1 region of the hippocampus were more severe in the BCAO group than in the BCAS group (P < 0.05; Figure 7).

When the central nervous system is damaged, astrocytes rapidly divide and proliferate, forming a glial scar for repair. In the BCAO group, the proliferation of astrocytes became apparent and the number of neurons was decreased, although not significantly different compared to the control group. Compared with the extent of neuronal damage in the BCAS group and the massive proliferation of astrocytes, the effects in the BCAS group were less severe after chronic cerebral hypoxia induced by hypoperfusion due to bilateral carotid stenosis (Figure 7).

## Discussion

The chronic cerebral hypoxia model established in this study has a low mortality rate and is easy to perform. The CBF was continually decreased for 2 weeks, and mild cognitive dysfunction was still observed. Simultaneously, glial cell activation, white matter lesions, and hippocampal cell damage were detected. These results suggest that this model can be used to study the mechanisms of VCI development.

Blood vessel stenosis induces chronic hypoxia and cognitive impairment in the brain (Steinback and Poulin, 2016). Different degrees of cognitive impairment have different clinical manifestations in patients with cognitive impairments such as dementia and mild cognitive impairment (MCI) (Langa and Levine, 2014; Qiu et al., 2018). The risk factors of VCI and MCI include age, carotid plaque, and hypertension, among others (Smith, 2017). The carotid plaque prevalence is 41.5%, and the carotid intima-media thickness increases by 1.66 × 10^–3^ mm annually (Mosing and Lundholm, 2018). The clinical manifestation of a carotid stenosis apart from an ischemic stroke (25–30%) is mainly asymptomatic carotid stenosis (ACS), which was once regarded as “silent” indicating the lack of a stroke or a brain hemorrhage. However, ACS is not “silent”. It is often considered to be the manifestation of normal aging processes due to the lack of clinical testing and scale assessments; thus, ACS is easily overlooked by patients and clinicians (Ryan et al., 2018). It is of great clinical significance to robustly identify patients with ACS and cognitive impairments and to determine the underlying mechanism of cognitive deterioration. Previous studies have confirmed that cognitive deterioration in patients with ACS precedes clinical treatment (i.e., severe carotid stenosis: 70–90% of all stenoses; (Sprung et al., 2016).

The original design idea of this study was conceived by a group of clinical anesthesiologists. They aimed to investigate whether cognitive function changes in asymptomatic patients with BCAS (Zhong et al., 2011; Yue et al., 2016) can be studied in animal models. This model should also be applicable to screen anesthetic compounds that reduce secondary damage in these patients (Tang et al., 2014). Hence, operability, repeatability, and a high survival rate in this model are critical.

The existing brain hypoperfusion models (Venkat et al., 2015) lack concomitant vascular disease risk factors and causative small vessel changes that may alter blood flow and cerebral autoregulation which can be impaired when hypoperfusion is induced in aged rats with BCAS. As the elderly population continues to age, the incidence of vascular cognitive impairment due to loss of vasodilatory functions increases (Uchida et al., 2000). In the present model, 18-month-old rats were selected because they offer more similarities to human than the 10- to 12-week-old animals used in most models (Venkat et al., 2015). Kitamura et al. (2016) and others used amberoid constrictors to induce a complete occlusion of the carotid artery instead of retaining a small residual blood flow resulting in a blood flow similar to that in the present model (Kitamura et al., 2016). In comparison to Jawhar et al. (2012) using the 5XFAD mouse model of Alzheimer’s disease and the C57BL/6 mouse model used by Shitaba et al., the rat model in the present study has a stronger ability for CBF compensation due to the existence of the posterior communicating artery and the intact basilar artery ring, simulating better the human disease. Compared with the mouse model by Shitaba et al., the BCAS rat model has different applications. While their mouse model is suitable for assessments of cognitive function impairments and genetic analyses, our rat model is rather useful for drug screening and early VCI research. Moreover, instead of Alzheimer’s disease and amyloidosis used by Jawhar et al., our model is related to vascular cognition, the cause of and white matter ischemia and cognitive impairment.

In our preliminary experiments (Please refer to the Supplementary Material for details), significant differences in cognitive dysfunction were identified in three age groups of rats with BCAS surgery, among others. The discrepancies could be attributed to the age of the BCAS rats, and the most obvious change in cognitive function was observed in the group of aged rats (18 months old). This result is in agreement with carotid stenosis caused by carotid atherosclerosis leading to chronic hypoxia and cognitive dysfunction in the human brain, mostly occurring in the elderly (Gupta et al., 2016). The Morris water maze was used to evaluate spatial and visual learning and memory with adverse motivation to assess hippocampal memory deficits (Ohno et al., 2006; Venkat et al., 2015; Choi et al., 2016). Furthermore, the findings in the water maze test show that the degree of cognitive dysfunction in BCAS rats did not reach that of rats with BCAO because the difference in escape latency was notably less compared to the BCAS group (Venkat et al., 2015; Choi et al., 2016). This indicates that the BCAS model is consistent with an early stage of vascular cognitive impairment or MCI (Hanseeuw et al., 2016). However, the number of rats used in the behavioral tests was small. In the future, the number of rats should be increased to more than fifteen per group to provide a more significant conclusion in the behavioral tests. Additionally, we aim to include this testing in future experiments and continue to explore the mechanisms of vascular cognitive dysfunction using assessments such as the Barnes circular maze, a balance beam task, and gait analysis to observe motor deficits (Venkat et al., 2015; Choi et al., 2016).

In the present experiments, the carotid arteries of aged rats narrowed to a diameter of 0.45 mm. This constriction of the carotid artery was measured to be 30–40% after surgery, consequently reducing the CBF values to 70.33 ± 4.00%. On day 30, the CBF values nearly recovered through compensatory mechanisms while a hypoxic state was maintained in aged BCAS rats. However, the results of the Morris water maze experiments demonstrated that mild chronic hypoxia causes only subtle memory impairments in rats. The memory of the platform position was not lost, only the search time was prolonged; this is consistent with the definition of MCI. By contrast, the CBF values were significantly reduced in the BCAO group. In this group, the vertebral artery could not fully compensate for the reduced blood flow within 30 days after surgery. The daily activities of these rats decreased, and the obvious symptoms of dementia appeared.

According to the DTI data, progressive microstructural deficits were present in both BCAS and BCAO rats. Minor alterations in the microstructural integrity were present in BCAS rats. Only small unilateral clusters in the frontoparietal somatosensory cortex and the external capsule/entorhinal cortex presented slightly attenuated mean diffusivity or FA values, respectively. These changes were aggravated in dementia, with large bilateral clusters of increased mean diffusivity and decreased FA values in BCAO rats. An additional region of interest-based analysis further revealed progressive microstructural alterations of FA values in the hippocampi and cingulate cortices of BCAS and BCAO rats. This progression is in line with *ex vivo* findings in early stages of vascular cognitive impairment and dementia pathology in this rat model as shown by us and others (Rorabaugh et al., 2017), including progressive amyloid plaque loads in various regions and increased microgliosis in the hippocampus. The histopathology as described here is in good agreement with previous reports of vascular cognitive impairment pathology in the BCAO model (Bazzigaluppi et al., 2018a, b) and could affect tissue organization, leading to altered diffusion measurements. More specifically, the pathology in the rat model of vascular cognitive impairment was reflected by increased mean diffusivity and decreased anisotropy; this is thought to represent microstructural cellular damage, alterations in tissue organization, the breakdown of diffusion barriers, and eventually neuronal loss (Weston et al., 2015). Crucially, as a stage preceding dementia, the DTI results in BCAS rats in the present study coincide with vascular changes in predementia patients.

Astrocyte proliferation has already been described in BCAS rats. When the central nervous system is damaged, astrocytes rapidly proliferate and repair the brain, forming glial scars (Marina et al., 2016). These results show that BCAS rats present hippocampal damage and repair, although the damage is more substantial in BCAO rats. Furthermore, glial activation indicates blood-brain barrier deficits and repair (Zenaro et al., 2017), and cognitive dysfunction is relative to the blood-brain barrier damage (Hattori et al., 2014).

Based on the present experimental results, there are significant differences between BCAS and BCAO rats. The former can be used as a new model of chronic cerebral hypoxia in terms of cerebral hypoxia time, changes in the white matter fiber structure, and hippocampal neuronal and vascular alterations to study the pathological processes in the early stages of VCI. Additionally, the BCAS rat model meets the criteria for establishing an MCI rat model characterized by old age, subtle memory impairments, mild neuropathological changes, changes in the cholinergic system, cerebrovascular alterations, and normal motor activity and feeding behavior (Pepeu, 2004). According to the microstructure of the rat hippocampal CA1 region, neuronal changes in this region were in agreement with mild cognition dysfunction, which accords with the established criteria of the MCI model (Pepeu, 2004). The present results also showed that the rat brain was in a persistent state of mild hypoperfusion for at least 14 days after surgery in MCI rats. Neuronal cells are sensitive to hypoxia that subsequently influences energy metabolism, decreases glucose utilization, and leads to abnormal protein synthesis and neuronal damage. The next criterium is the change in the cholinergic system. For this aspect, past studies have reported that aged rats (>18 months) exhibit decreased acetylcholine release from cerebral cortex, hippocampus, and striatum cells (Lindner et al., 1992; Bizon et al., 2012). In summary, the cerebral hypoperfusion in rats induced by BCAS surgery accords with the requirements for an MCI animal model.

This study was not only designed to validate a model of vascular cognitive impairment but also to explore the underlying pathological processes. Thus, the GABA_A_R-α1 and hnRNPA2/B1 expression levels, as well as the proliferation of hippocampal astrocytes, in rats at 30 days after BCAS and BCAO surgery were examined. The expression of GABA_A_R-α1 was significantly decreased in rats with MCI and those with dementia. The GABA_A_R-α1 subunit is widely distributed in the mammalian brain where it regulates inhibitory neurotransmission in the central nervous system and the sedative-hypnotic effects of general anesthetics including isoflurane and propofol. Mutations in the transmembrane domain of the GABA_A_R-α1 subunit affect the pharmacological effects of propofol and isoflurane (Ghosh et al., 2013).

Notably, hnRNPA2/B1 is an important transcription factor that regulates energy metabolism and protein translation (Guha et al., 2016; Hutten and Dormann, 2016). It is a crucial target of the mitochondrial retrograde signaling system (Zhao et al., 2018) and is involved in the activation of GABA_A_R-α messenger RNA transcription (Labrakakis et al., 2014; Ryan et al., 2018). The expression of hnRNPA2/B1 is elevated in patients with pre-Alzheimer’s disease, whereas the expression of hnRNPA2/B1 in neuronal cells with a severe protein metabolism imbalance is decreased (Mizukami et al., 2005). In the present study, hnRNPA2/B1 expression was significantly decreased in the hippocampus of MCI rats but increased in animals after BCAO surgery. Therefore, it is hypothesized that the decrease in hnRNPA2/B1 expression can be used as another screening criterium to validate MCI models, except for mild cognitive dysfunction testing in cognitive function impairment.

Furthermore, in this experiment, we explored the differences between the BCAS and BCAO models and tried to explain these differences with respect to microcirculatory dysfunction. Cerebral microcirculatory dysfunction is one of the common pathologies associated with vascular cognitive impairment (De Silva and Faraci, 2016), which causes changes in white matter lesions (Iadecola et al., 2019) and cognitive dysfunction (Thorin-Trescases et al., 2018). For example, Kitamura et al. used spontaneously hypertensive rats as a model of early-stage cerebral small vessel disease. They demonstrate that hypertension-induced small vessel degeneration and endothelial cell damage cause microcirculation disorders that eventually lead to white matter damage and cognitive dysfunction (Kitamura et al., 2016).

We hypothesize that the BCAS and BCAO models in this study are also accompanied by microcirculatory disorders. One of the reasons is the decrease in cerebral blood flow caused by stenosis or occlusion of the carotid arteries, resulting in energy metabolism dysfunction of glial, endothelial, and neuronal cells (Ogoh, 2017). According to our transmission electron microscopy results, the mitochondrial ridge breaks in the BCAS group, more apoptotic bodies appear in the BCAO group, and the expression of hnNRPA2, a protein involved in mitochondrial energy metabolism (Guha et al., 2016), changes in the BCAS group, confirming our hypothesis.

However, the mechanisms of white matter damage caused by microcirculation disorder differ slightly between BCAS and BCAO models. In the BCAO group, the cerebral blood flow presents a sharp decline leading to acute ischemic impairment in cerebral microcirculation, acute cerebral ischemic infarction, and high mortality rates (Naritomi, 1991) regardless of compensation via vertebral arteries and collateral circulation that typically contributes to CBF recovery (Kato et al., 2019). Thus, we deduce that BCAO rats are more akin to models of acute focal cerebral ischemia caused by middle cerebral artery occlusion (Naritomi, 1991; Shah et al., 2019). This may explain the differences in FA value changes between the BCAS and BCAO models. In the current study, regions of increased and decreased FA values coexisted simultaneously in the brains of BCAO rats. Prior studies (Pitkonen et al., 2012; Mayo et al., 2018) found that an increase in FA value exists in the acute phase of cerebral infarction, whereas a decrease in FA value indicates white matter or nerve fiber bundle damage. Therefore, we suggest that the BCAO model corresponds to a simultaneous presence of smaller infarcts from acute ischemia and white matter damage, whereas the FA values in the BCAS group are solely decreased indicating slight white matter damage.

It should be noted that due to the presence of collateral circulation and vertebral arteries, the reduction in cerebral blood flow in the carotid arteries of the BCAS group was not sufficient to cause severe microcirculation dysfunction, which in turn caused the limited cognitive impairment in spatial memory in adult rats (12 months). We observed this phenomenon in previous experiments. However, in our experiments, the cerebral blood flow in the BCAS rats slowly recovered to 90% of the baseline within 15 days, but the cognitive impairment still existed in 18-month-old rats. In our opinion, the main reason for this observation is the age of the animals. Similar to the spontaneously hypertensive rat model, normal aging can also cause vasodilatory dysfunctions (Toth et al., 2017; Yang et al., 2017). Published studies (De Silva and Faraci, 2016) aging can also lead to energy transmission dysfunction in astrocytes, which causes dysfunction of endothelial cells, affects vasoconstriction, and modulates compensatory microcirculation along the blood-brain barrier. Our GFAP staining results support our hypothesis that in BCAS rats, glial cells are activated, whereas neurons are still slightly damaged.

Therefore, we believe that in the BCAS model, a combination of energy metabolism disorder and aging-induced vasodilatory dysfunction caused by CBF decreases lead to impaired microcirculatory functions and subsequently to cognitive dysfunction (Santisteban and Iadecola, 2018). The pathological cascade is characterized by activation of astrocytes, neuronal damage, and ultimately white matter damage.

MCI is a transitional state between dementia and normal aging, with a high risk of conversion to dementia, but the degree of memory impairment does not affect normal life activities. Importantly, MCI impairments are reversible (Sanford, 2017). At present, propofol and sevoflurane, among other drugs, influence cognitive functions via the cholinergic system (Logginidou et al., 2003; Wang et al., 2018). Also, whether the process aggravates the damage in MCI patients has not been clarified yet. A key issue is the rational use of drugs and the safe screening of new drugs in MCI patients. Therefore, recent developments in MCI and dementia assessments have emphasized the need for animal models of vascular MCI induced by chronic cerebral hypoperfusion with low mortality, simplicity, and high stability. Based on previous MCI animal models, Pepeu suggested five criteria for their establishment and screening. To explore the development and mechanisms in vascular cognitive impairment and to examine the differences between dementia and MCI, the classic vascular cognitive impairment model was included in this study to compare the differences between the two models, providing further evidence for an MCI diagnosis.

## Conclusion

In conclusion, severe bilateral carotid stenosis induced mild cognitive dysfunction and changes in the microscopic structure of the brain in aged rats. The chronic hypoxia model was established successfully; this model could be used to further explore vascular cognitive impairment.

## Ethics Statement

The study was reviewed and approved by the Institutional Animal Care and Use Committee of Tianjin Medical University. Rats were cared for according to the guidelines of the National Institutes of Health Guide and the United Kingdom Animals (Scientific Procedures) Act (1986) for Care and Use of Laboratory Animals.

## Data Availability Statement

All datasets generated for this study are included in the article/Supplementary Material.

## Author Contributions

HW and MZ contributed the included funding acquisition, provided the resources, wrote, reviewed, and edited the manuscript. JW contributed to the methodology, wrote the original draft, and curated data. CY and JM processed the data. DL, TL, YS, ZY, and WH conducted formal analyses. All authors may have one or more contributory role.

## Conflict of Interest

The authors declare that the research was conducted in the absence of any commercial or financial relationships that could be construed as a potential conflict of interest.

## Footnotes

1 https://www.mristudio.org

2 https://www.fil.ion.ucl.ac.uk/spm/software/spm8/

3 https://imaris.oxinst.com/

4 https://imagej.net/Fiji
