# Supplementary material for: A New Rat Model of Chronic Cerebral Hypoperfusion Resulting in Early-Stage Vascular Cognitive Impairment
Source: Front Aging Neurosci. 2020 Apr 15;12:86. doi: 10.3389/fnagi.2020.00086 (PMC7174718; doi:10.3389/fnagi.2020.00086)
Supplement: Supplementary file 1 [file Table_1.DOCX]

Supplementary Material

# Preliminary Experiments

**Surgical Procedures in the Rat Model of Bilateral Common Carotid Artery (CCA) Stenosis (BCCS)** **in different age groups**

The rat model of cerebral hypoperfusion was established in different age groups. Young (4 months; n = 6/11; 150 ± 50 g), middle-aged (12 months; n = 6/6; 300 ± 50 g), and aged (18 months; n = 6/7; 500 ± 50 g) male Wistar rats underwent bilateral carotid artery stenosis surgery before test firstly. then trained and tested in an acquisition water maze task, get with control rat in each age group. Every age group had its age-matched sham-operated control group.

After intraperitoneal (i.p.) injection of 10% thiotetrabarbital (100 mg/kg), a midline neck incision was made to expose and dissociate the bilateral CCAs. A syringe needle (0.15 mm, 0.25 mm, or 0.45 mm in diameter. We used color Doppler ultrasonography to select a suitable steel needle diameter that can achieve postoperatively a 70% carotid stenosis.) was then tied tightly to the CCA at 1.5 cm from the bifurcation of the internal and external carotid arteries to six rats in every age group. Additional six control rats in every age group were sham-operated, i.e., the bilateral CCAs were only exposed and dissociated. After ensuring that the slipknot was firmly fixed, the needle was carefully removed, and the wound was sutured. Thirty days after surgery, the Morris water maze test was performed to detect cognitive dysfunction in rats with cerebral hypoperfusion and sham-operated rats.

The results of the place navigation experiment showed that the time required to reach the platform (average escape latency) was longer in the group of aged rats compared to their control group (P < 0.05) on the second to fifth day after surgery. By contrast, there were no significant differences between the groups of young or middle-aged rats and their corresponding control groups.

| **Table** A Comparison of average escape latencies in the place navigation test among the three age groups. | | | | | | | | | |
| --- | --- | --- | --- | --- | --- | --- | --- | --- | --- |
|  |  | young group  (4 months) | |  | middle-aged group  (12 months) | |  | aged group  (18 months) | |
| group | n | operation | control |  | operation | control |  | operation | control |
| day2 | 6 | 17.65±6.45 | 14.00±2.83 |  | 12.62±3.67 | 11.93±2.26 |  | 39.09±3.05* | 29.1±6.13 |
| day3 | 6 | 8.73±2.45 | 7.59±1.64 |  | 7.20±1.97 | 5.71±1.55 |  | 16.89±4.69* | 13.76±4.33 |
| day4 | 6 | 1.33±1.33 | 2.25±2.25 |  | 1.44±1.44 | 0.79±0.79 |  | 1.77±1.77* | 1.39±1.39 |
| day5 | 6 | 6.20±1.57 | 6.03±1.01 |  | 6.58±2.01 | 5.24±1.59 |  | 11.9±1.80* | 9.39±1.17 |

Data are expressed as the mean ± SD (n = 6/group). *P < 0.05 compared with the control group; ^#^P < 0.05 compared with the BCAS group; ^P < 0.05 compared with the BCAO group.

| **TableB** Comparisons of the times over the hidden platform and percentages of the swimming time to the platform in the spatial probe test among the three age groups. | | | | | | | | | |
| --- | --- | --- | --- | --- | --- | --- | --- | --- | --- |
|  |  |  |  |  |  |  |  |  |  |
|  |  | young group  (4 months) | |  | middle-aged group  (12 months) | |  | aged group  (18 months) | |
|  | n | operation | control |  | operation | control |  | operation | control |
| percentage of swimming time to the platform in the spatial probe test | 6 | 18.25±2.09 | 18.14±3.82 |  | 18.03±2.48 | 19.99±3.72 |  | 38.56±2.97* | 27.43±2.29 |
| time over the hidden platform | 6 | 2.37±0.89 | 2.44±0.62 |  | 2.07±0.62 | 2.08±0.38 |  | 3.84±1.06* | 3.07±0.62 |

Data are expressed as the mean ± SD (n = 6/group). *P < 0.05 compared with the control group; ^#^P < 0.05 compared with the BCAS group; ^P < 0.05 compared with the BCAO group.
